# Prognostic value of the systemic immune-inflammation index in non-muscle invasive bladder cancer

**DOI:** 10.1007/s00345-021-03740-3

**Published:** 2021-06-18

**Authors:** Satoshi Katayama, Keiichiro Mori, Benjamin Pradere, Ekaterina Laukhtina, Victor M. Schuettfort, Fahad Quhal, Reza Sari Motlagh, Hadi Mostafaei, Nico C. Grossmann, Pawel Rajwa, Marco Moschini, Romain Mathieu, Mohammad Abufaraj, David D’Andrea, Eva Compérat, Martin Haydter, Shin Egawa, Yasutomo Nasu, Shahrokh F. Shariat, European Association of Urology–Young Academic Urologists Urothelial Carcinoma Working Group (EAU-YAU)

**Affiliations:** 1 Department of Urology, Medical University of Vienna, Währinger Gürtel 18-20, 1090 Vienna, Austria; 2 Department of Urology, Dentistry and Pharmaceutical Sciences, Okayama University Graduate School of Medicine, Okayama, Japan; 3 Department of Urology, The Jikei University School of Medicine, Tokyo, Japan; 4 Institute for Urology and Reproductive Health, Sechenov University, Moscow, Russia; 5 Department of Urology, University Medical Center Hamburg-Eppendorf, Hamburg, Germany; 6 Department of Urology, King Fahad Specialist Hospital, Dammam, Saudi Arabia; 7 Men’s Health and Reproductive Health Research Center, Shahid Beheshti University of Medical Sciences, Tehran, Iran; 8 Research Center for Evidence Based Medicine, Tabriz University of Medical Sciences, Tabriz, Iran; 9 Department of Urology, University Hospital Zurich, Zurich, Switzerland; 10 Department of Urology, Medical University of Silesia, 41-800 Zabrze, Poland; 11 Department of Urology, Luzerner Kantonsspital, Luzern, Switzerland; 12 Department of Urology, Rennes University Hospital, 2 Rue Henri le Guilloux, 35000 Rennes, France; 13 Division of Urology, Department of Special Surgery, The University of Jordan, Amman, Jordan; 14 Department of Urology, Landesklinikum Wiener Neustadt, Vienna, Austria; 15 Department of Urology, University of Texas Southwestern Medical Center, Dallas, TX USA; 16 Department of Urology, Second Faculty of Medicine, Charles University, Prague, Czech Republic; 17 Department of Urology, Weill Cornell Medical College, New York, NY USA; 18 Karl Landsteiner Institute of Urology and Andrology, Vienna, Austria

**Keywords:** SII, NMIBC, Survival, Biomarker, Intermediate-risk

## Abstract

**Purpose:**

We assessed the prognostic value of systemic immune-inflammation index (SII) to refine risk stratification of the heterogeneous spectrum of patients with non-muscle-invasive bladder cancer (NMIBC)

**Methods:**

In this multi-institutional cohort, preoperative blood-based SII was retrospectively assessed in 1117 patients with NMIBC who underwent transurethral resection of bladder (TURB) between 1996 and 2007. The optimal cut-off value of SII was determined as 580 using the best Youden index. Cox regression analyses were performed. The concordance index (C-index) and decision curve analysis (DCA) were used to assess the discrimination of the predictive models.

**Results:**

Overall, 309 (28%) patients had high SII. On multivariable analyses, high SII was significantly associated with worse PFS (hazard ratio [HR] 1.84; 95% confidence interval [CI] 1.23–2.77; *P* = 0.003) and CSS (HR 2.53; 95% CI 1.42–4.48; *P* = 0.001). Subgroup analyses, according to the European Association of Urology guidelines, demonstrated the main prognostic impact of high SII, with regards to PFS (HR 3.39; 95%CI 1.57–7.31; *P* = 0.002) and CSS (HR 4.93; 95% CI 1.70–14.3; *P* = 0.005), in patients with intermediate-risk group; addition of SII to the standard predictive model improved its discrimination ability both on C-index (6% and 12%, respectively) and DCA. In exploratory intergroup analyses of patients with intermediate-risk, the improved discrimination ability was retained the prediction of PFS and CSS.

**Conclusion:**

Preoperative SII seems to identify NMIBC patients who have a worse disease and prognosis. Such easily available and cheap standard biomarkers may help refine the decision-making process regarding adjuvant treatment in patients with intermediate-risk NMIBC.

**Supplementary Information:**

The online version contains supplementary material available at 10.1007/s00345-021-03740-3.

## Introduction

The standard treatment for non-muscle-invasive bladder cancer (NMIBC) is transurethral resection of bladder (TURB) followed by intravesical instillation chemotherapy or immunotherapy, according to individual patient risk for disease recurrence and progression [1]. Despite complete resection and adjuvant intravesical instillation therapy, approximately 70% of these patients experience disease recurrence and 30%, eventually, experience progression [2, 3]. Several prognostic models and biomarkers have been investigated as predictors of oncologic outcomes to guide clinical decision-making and patient counselling [4, 5]. However, none of them have achieved sufficient accuracy to be integrated into daily routine clinical practice [1, 2, 6–8].

The immune system, including the inflammatory response and the tumor microenvironment, plays an important role in the clinical and biological behavior and outcomes of bladder cancer (BC) [9]. The systemic immune-inflammation index (SII), an immune and inflammatory index based on neutrophil, lymphocyte, and platelet counts, has been shown to be associated with oncological outcomes in several types of cancer [10, 11].

While SII has already been reported to be of prognostic value in MIBC and high-risk NMIBC patients, its role in NMIBC patients with intermediate-risk remains unclear [12, 13]. Given BC, which is an inflammatory disease, has efficiently been treated using bacillus Calmette–Guerin (BCG), an agent that is known to trigger a good strong inflammatory and immunologic response. We hypothesized that the simple easily available SII can help classify NMIBC patients according to their individualized risk of recurrence and progression, especially in patients with intermediate-risk [14]. Toward this aim, we explored the prognostic significance of preoperative SII in a large multi-institutional cohort of NMIBC patients.

## Materials and methods

### Patient selection

Our study included 1117 consecutive patients treated with TURB for primary or recurrent NMIBC with or without adjuvant intravesical instillation therapy at four academic centers in US and Europe between 1996 and 2007 [7]. All patients were histologically confirmed to have urothelial carcinoma with only minor (less than 10%) involvement of variant components, if any. None of the patients had metastatic disease, concurrent upper tract urothelial or urethral cancer invasion at the time of TURB. This study obtained institutional review board approval at each participating institution. All sites agreed to institutional data sharing prior to study initiation.

### Data collection and pathologic evaluation

Pretreatment blood-based SII values were assessed within the 30 days prior to TURB. SII was calculated as platelet count × neutrophil/lymphocyte count. SII and demographic, pathologic, and survival outcomes data were collected and entered into a computerized database. The optimal SII cut-off value was defined by creating a time-dependent receiver operating characteristic (ROC) curve to yield the highest Youden index value. Using this score the overall population was divided into two separate SII groups (> 580 vs. ≤ 580). The specimens were reviewed at the beginning of the study at each center by expert genitourinary pathologists. They were blinded to the previous history of the patient and to the clinical development after specimen acquisition. The pathologic stage was reassigned using the 2010 American Joint Committee on Cancer TNM staging system and tumor grade according to the 1973 World Health Organization (WHO) grading system. Patients were categorized into low, intermediate, and high (added highest only for progression risk classification) risk groups for the prediction of disease progression and recurrence according to the European Association of Urology (EAU) guidelines and European Organization for Research and Treatment of Cancer risk tables (EORTC) [1, 15, 16].

### Management and follow-up

All patients underwent complete TURB. A second-look resection was performed 2–6 weeks after initial treatment based on the pathologic and intraoperative findings according to the guidelines at the time. In general, a second look was indicated in cases of incomplete initial TURB or doubt about the completeness of a TURB; if there was no muscle in the specimen after initial resection, with the exception of Ta LG/G1 tumors and primary CIS; in T1 tumors. Immediate single-dose postoperative instillation chemotherapy, adjuvant intravesical chemotherapy, or adjuvant BCG immunotherapy were administered according to risk categories of disease recurrence and progression based on current guidelines.

All patients were generally followed up according to the EAU guidelines. This included urinary cytology and a cystoscopy every 3 months for the first 2 years after surgery; after that, every 6 months for 3 years, and then, annually. Disease recurrence was defined as the first tumor relapse in the bladder regardless of tumor stage. Disease progression was defined as tumor relapse in the bladder with tumor stage T2 or higher. Disease occurrence in the upper urinary tract was considered as a second primary.

### Statistical analysis

Associations of SII with categorical variables were assessed using chi-squared tests and differences in continuous variables were analyzed using Mann–Whitney *U* tests. Recurrence-free (RFS), progression-free (PFS), cancer-specific (CSS), and overall (OS) survival were graphically visualized using the Kaplan–Meier method. Differences between groups were assessed with the log-rank test. Multivariable Cox regression models were used to investigate the associations of SII with each survival outcome. The discrimination of the model was evaluated using the Harrel’s concordance index (C-index). The additional clinical net-benefit of SII was evaluated using decision curve analysis (DCA). In addition to subgroup analyses based on patients with intermediate-risk, we performed exploratory analyses of patients with intermediate-risk based on the risk stratification proposed by the International Bladder Consultation Group (IBCG) [17, 18]. In these analyses, we excluded patients who received intravesical BCG therapy due to the changes of treatment strategies depending on the previous BCG therapy. All *P* values were two-sided, and statistical significance was defined as *P* < 0.05. Statistical analyses were performed using R version 3.6.3 (R Foundation for Statistical Computing, Vienna, Austria) and Stata/MP 14.2 statistical software (Stata Corp., College Station, TX, USA).

## Results

Of the 1117 patients included in this study, 309 (28%) were categorized into the high SII (SII > 580) group and 808 (72%) into the low (SII ≤ 580). Patients with low-, intermediate-, high-, and very high-risk disease were 18%, 37%, 41%, and 4%, respectively. The associations of SII with standard clinicopathologic characteristics are shown in Table 1. There was no significant difference between groups, apart from sex (*P* = 0.048).

**Table 1:** Patients’ characteristics according to the peripheral SII level (low vs high) in patients with NMIBC treated by TURB

	ALL	Low SII	High SII	*P*
Total, *n* (%)	1117	808 (72)	309 (28)	
Gender, *n* (%)				**0.048**
Male	855 (76.5)	631 (78)	224 (72)	
Female	262 (23.5)	177 (22)	85 (28)	
Age, median (IQR)	67(58–74)	67(59–74)	65(56–73.2)	0.07
Prior recurrence, *n* (%)				0.781
Primary	931 (83)	675 (84)	256 (83)	
Recurrent	186 (17)	133 (16)	53 (17)	
Tumor stage, *n* (%)				0.41
pTa	653 (58)	464 (57)	189 (61)	
pTis	21 (2)	17 (2)	4 (1)	
pT1	443 (40)	327 (41)	116 (38)	
Tumor grade, *n* (%)				0.63
G1	231 (21)	169 (21)	62 (20)	
G2	398 (36)	281 (35)	117 (38)	
G3	488 (44)	358 (44)	130 (42)	
Concomitant CIS, *n* (%)				0.44
Yes	66 (6)	45 (6)	21 (7)	
No	1051 (94)	763 (94)	288 (93)	
Smoking, *n* (%)				0.27
No	272 (24)	205 (25)	67 (22)	
Yes	845 (76)	603 (75)	242 (78)	
Tumor size, *n* (%)				0.19
< 3	816 (73)	599 (74)	217 (70)	
≥ 3	301 (27)	209 (26)	92 (30)	
Number of tumors, *n* (%)				0.06
Single	718 (64)	534 (66)	184 (60)	
2–7	297 (27)	199 (24)	98 (31)	
8 ≤	102 (9)	75 (9)	27 (9)	
Adjuvant intravesical therapy, *n* (%)				0.87
No intravesical therapy	624 (56)	457 (57)	167 (54)	
Adjuvant BCG	300 (27)	212 (26)	88 (29)	
Adjuvant chemotherapy	48 (4)	35 (4)	13 (4)	
Early single instillation	145 (13)	104 (13)	41 (13)	
EAU risk for progression, *n* (%)				0.99
Low	195 (17)	140 (17)	55 (18)	
Intermediate	415 (37)	302 (37)	113 (37)	
High	463 (42)	335 (42)	128 (41)	
Very high	44 (4)	31 (4)	13 (4)	
EORTC risk for recurrence, *n* (%)				0.16
Low	141 (13)	99 (12)	42 (14)	
Intermediate	936 (84)	685 (85)	251 (81)	
High	40 (4)	24 (3)	16 (5)	

Bold P values are considered statistically significant

*NMIBC* non-muscle invasive bladder cancer, *TURB* transurethral resection of bladder, *SII* systemic immune-inflammation index, *CIS* carcinoma in situ, *BCG* bacillus Calmette–Guerin *EAU* the European Association of Urology, *EORTC* the European Organization for Research and Treatment of Cancer risk tables

### Association with survival outcomes in the entire cohort

During the median follow-up of 64 months (IQR 26–110 months), 470 (42.1%) patients experienced disease recurrence, 103 (9.2%) experienced disease progression, and 299 (27%) died from any causes, and 50 (4.5%) died from BC. In Kaplan–Meier analyses, patients with high SII were at significantly increased risk of worse PFS and CSS (hazard ratio [HR] 1.75; 95% confidence interval [CI] 1.17–2.60; *P* = 0.005, and HR 2.32; 95% CI 1.33–4.05; *P* = 0.002, respectively), but not RFS and OS (HR 1.21; 95% CI 0.99–1.47; *P* = 0.06, and HR 0.95; 95% CI 0.73–1.23; *P* = 0.70, respectively) (Supplementary Fig. 1). On multivariable Cox regression analyses that adjusted for the effects of established confounders, pre-TURB SII remained associated with both PFS and CSS (HR 1.84; 95% CI 1.23–2.77; *P* = 0.003, and HR 2.53; 95% CI 1.42–4.48; *P* = 0.001, respectively) (Table 2).

**Table 2:** Multivariable Cox regression analyses predicting survival outcomes

	OS			CSS			PFS			RFS		
	Multivariable			Multivariable			Multivariable			Multivariable		
	HR	(95% CI)	*P* value	HR	(95% CI)	*P* value	HR	(95% CI)	*P* value	HR	(95% CI)	*P* value
Age (ref. ≤ 70)												
> 70	3.13	2.48–3.96	** < 0.001**	1.99	1.12–3.54	**0.019**	1.89	1.27–2.81	**0.002**	1.72	1.43–2.08	** < 0.001**
Sex	0.74	0.56–0.99	0.045	0.78	0.40–1.54	0.47	1.06	0.68–1.65	0.81	0.94	0.76–1.17	0.60
Smoking	0.92	0.70–1.20	0.52	1.79	0.80–4.01	0.16	1.96	1.11–3.46	**0.020**	1.10	0.88–1.37	0.40
Adjuvant BCG	0.79	0.58–1.09	0.16	0.84	0.43–1.65	0.62	0.69	0.43–1.13	0.14	0.84	0.67–1.05	0.12
Concomitant CIS	0.99	0.52–1.86	0.97	0.97	0.31–2.98	0.95	0.97	0.42–2.21	0.94	0.88	0.58–1.34	0.55
Number of tumor (ref. < 8)												
8 ≤	1.67	1.11–2.52	**0.014**	2.80	1.34–5.85	**0.006**	2.14	1.25–3.67	**0.006**	1.03	0.73–1.45	0.87
Tumor size (ref. < 3)												
3 ≤	1.10	0.84–1.41	0.51	0.92	0.51–1.67	0.78	1.06	0.70–1.61	0.78	2.38	1.96–2.88	** < 0.001**
Prior recurrence rate (ref. Primary, ≤ 1 recurrence/yr)												
1 < recurrence/yr										1.09	0.76–1.55	0.65
Grade (ref. Grade 1)												
Grade 2	1.29	0.93–1.76	0.13	11.6	1.54–87.0	**0.017**	2.49	1.15–5.42	**0.021**	1.74	1.33–2.29	** < 0.001**
Grade 3	1.57	0.79–3.12	0.20	42.7	4.99–366.3	**0.001**	8.83	3.37–23.1	** < 0.001**	2.50	1.58–3.95	** < 0.001**
Stage (ref. pTa, Tis)												
pT1	0.77	0.40–1.46	0.42	0.36	0.15–0.90	0.03	0.42	0.21–0.83	**0.013**	0.42	0.28–0.63	** < 0.001**
SII	1.04	0.80–1.36	0.78	2.53	1.42–4.48	**0.001**	1.84	1.23–2.77	**0.003**	1.23	1.00–1.50	**0.046**
C-index without SII	0.66			0.75			0.71			0.66		
C-index with SII	0.67			0.78			0.74			0.66		

Bold P values are considered statistically significant

*BCG* bacillus Calmette–Guerin, *CIS* carcinoma in situ, *SII* systemic inflammatory index, *OS* overall survival, *CSS* cancer-specific survival, *PFS* progression-free survival, *RFS* recurrence-free survival

### Subgroup analyses in patients with intermediate-risk NMIBC

In patients with intermediate-risk NMIBC, high SII was significantly associated with an increased probability of worse PFS, and CSS (HR 3.39; 95% CI 1.57–7.31; *P* = 0.002, and HR 4.93; 95% CI 1.70–14.3; *P* = 0.005, respectively), but not with RFS and OS (HR 1.40; 95% CI 1.00–1.96; *P* = 0.05, and HR 1.21; 95% CI 0.79–1.84; *P* = 0.38) (Table 3). Addition of pre-TURB SII levels to a basic model based on age, sex, tumor size, multifocality, tumor grade, and tumor stage increased the discriminatory ability for the prediction of both PFS (change of C-index 6%) and CSS (change of C-index 12%). DCA showed that adding SII to a standard model increased the net benefit for the prediction of PFS and CSS (Supplementary Fig. 2).

**Table 3:** Multivariable cox regression analyses in 336 patients with intermediate-risk who did not receive intravesical BCG therapy

	OS			CSS			PFS			RFS		
	Multivariable			Multivariable			Multivariable			Multivariable		
	HR	(95% CI)	*P* value	HR	(95% CI)	*P* value	HR	(95% CI)	*P* value	HR	(95% CI)	*P* value
Age (ref. ≤ 70)												
> 70	2.20	1.54–3.16	** < 0.001**	1.11	0.34–3.64	0.86	1.89	0.86–4.15	0.11	1.52	1.11–2.08	**0.009**
Sex	0.72	0.46–1.14	0.16	0.66	0.18–2.37	0.54	0.52	0.20–1.37	0.19	0.65	0.45–0.95	**0.027**
Smoking	1.04	0.68–1.61	0.84	3.23	0.61–17.1	0.17	2.67	0.89–7.96	0.08	1.28	0.89–1.85	0.18
Number of tumor (ref. < 8)												
8 ≤	1.28	0.56–2.94	0.57	5.62	1.14–27.8	**0.039**	3.56	1.20–10.6	**0.022**	1.17	0.65–2.12	0.61
Tumor size (ref. < 3)												
3 ≤	1.05	0.72–1.54	0.80	1.13	0.36–3.54	0.57	1.17	0.52–2.63	0.70	2.09	1.52–2.87	** < 0.001**
Prior recurrence rate (ref. Primary, ≤ 1 recurrence/yr)												
1 < recurrence/yr										1.36	0.67–2.78	0.39
Grade (ref. Grade 1, 2)												
Grade 3	1.29	0.31–5.37	0.73	13.9	2.37–81.3	**0.007**	12.1	3.75–38.9	** < 0.001**	1.10	0.44–2.77	0.84
SII	1.21	0.79–1.84	0.38	4.93	1.70–14.3	**0.005**	3.39	1.57–7.31	**0.002**	1.40	1.00–1.96	0.05
C-index without SII	0.61			0.72			0.67			0.63		
C-index with SII	0.62			0.84			0.73			0.63		

Bold P values are considered statistically significant

*BCG* bacillus Calmette–Guerin, *SII* systemic inflammatory index, *OS* overall survival, *CSS* cancer-specific survival, *PFS* progression-free survival, *RFS* recurrence-free survival

In exploratory analyses for intermediate-risk patients with 1–2 factor determined according to the algorithm proposed by IBCG, high SII was significantly associated with worse PFS and CSS (HR 3.71; 95% CI 1.62–8.51; *P* = 0.002, HR 5.15; 95% CI 1.72–16.9; *P* = 0.005, respectively) (Supplementary Table 1).

## Discussion

In this large multi-institutional study, we investigated the clinical value of the preoperative blood-based SII for patients with primary or recurrent NMIBC treated with TURB. We found that high SII was an independent predictive factor for PFS and CSS, but not for OS in NMIBC. Subgroup analyses demonstrated that in intermediate-risk patients, high SII was a significant predictor of PFS, and CSS; furthermore, for patients with 1–2 factors substantified by the IBCG algorithm, high SII remained also an independent predictor of PFS and CSS. These findings suggest that SII would help refine our clinical practice by helping identify individualized treatment strategies in the large cohort of heterogeneous patients with intermediate-risk BC.

Inflammatory conditions induced by mediators such as chemokines or cytokines extrinsically aid proliferation and survival of malignant cells, angiogenesis, and metastasis; while the activation of oncogene drives intrinsic inflammatory pathways. Thus, inflammation and cancer are strongly linked, and inflammation can create the nurturing environment for cancer formation and promotion [14, 19]. To date, the wide spectrum of biological behavior in NMIBC, especially in intermediate-risk disease, has encouraged researchers to explore several tissue- and blood-based biomarkers [20]. Of these, the prognostic values of blood-based biomarkers measuring systemic inflammatory responses have been investigated in various malignancies, driven by advantages in availability and cost. Recently, SII, determined by multiplying NLR by platelet count demonstrated promising discriminatory power for cancer progression with several functional mechanisms having been proposed. Neutrophils, a hallmark of inflammation, interact with circulating tumor cells (CTCs) facilitating binding to the endothelium. This interaction can promote tumor progression and metastasis by inducing tumor cell proliferation, stimulating angiogenesis, and suppressing the function of the adaptive immune response in the tumor microenvironment [21, 22]. Impairment of immune cells triggered by the evolving tumor microenvironment can be detected in cancer patients [23]. Aggregated active platelets envelop around CTCs (present in 18–20% of patients with NMIBC and associated with inferior oncologic outcomes), shielding CTCs from immunological attacks in high-risk NMIBC (especially pT1 G3 disease) [24, 25]. Taken together, SII objectively reflects a combination of the inflammatory and host immune system status.

In our study, we confirmed the significant prognostic value of SII in NMIBC, regardless of the risk category. These findings suggest that low SII level may help identify patients who could benefit from meticulous surveillance instead of additional treatments which are often associated with adverse effects (e.g., maintenance therapy following induction intravesical BCG or chemotherapy).

Moreover, subgroup analyses revealed that in intermediate-risk NMIBC patients who did not receive BCG therapy, addition of SII to a basic model improved the discrimination power for the prediction of PFS (6%) and CSS (12%), along with the improved net benefit within the clinical reasonable range of thresholds in the DCA. Additionally, the impact of SII in this group was greater than that in the overall analyses (nearly five-times higher risk for CSS and more than three-times higher risk for PFS). Although the definition of intermediate-risk was different from this study, we and others have previously highlighted the heterogeneity of the intermediate-risk NMIBC group [17, 18]. Previous studies suggested that BCG therapy with maintenance was superior to BCG induction only or chemotherapy induction with maintenance for preventing recurrence or progression in patients with intermediate-risk NMIBC [26, 27]. However, given the heterogeneity of this population, variety of available options, and adverse effects of BCG, urologists have often been unsure about the type, schedule, and length of adjuvant therapy, resulting in low compliance with NMIBC guidelines [28]. Our findings suggest that pre-therapy SII would help guide individual treatment strategies of patients with intermediate-risk NMIBC, leading to improved outcomes and compliance with guidelines.

Moreover, due to concerns regarding the large heterogeneity in the risk of recurrence and progression in NMIBC patients with intermediate-risk, the IBCG proposed an intergroup subclassification in patients with intermediate-risk disease. However, for patients with 1–2 factors according to the IBCG algorithm, urologists still have a choice of regimen in adjuvant therapy. We confirmed that for patients with 1–2 factors, SII remained an independent factor for the prediction of both PFS and CSS with considerable increases in C-index (7% and 12%, respectively). Thus, pre-TURB SII can help guide decision of treatment regimen in intermediate-risk NMIBC patients with 1–2 factors (for example, chemotherapy for patients with low SII, and BCG for those with high SII).

Several limitations of our study should be acknowledged. First, due to the retrospective and multi-institutional aspect, therapeutic strategies and pathologic indications varied among the institutions, which may have affected survival outcomes. Second, we did not capture comorbidities at the time of SII measurement, which are major confounding factors due to their strong effects on the value of each variable. Third, we lack data on prior intravesical therapy, radical cystectomy, and variant histology, which could have influenced the oncologic outcomes. Finally, SII was measured preoperatively and the lack of standardized cut-off points. As such, measuring the postoperative SII might have some additive prognostic value.

## Conclusion

We found that high pre-therapy SII was an independent predictive factor for both PFS and CSS in patients with NMIBC; this impact was enhanced in patients with intermediate-risk. Due to the improvement to the discriminatory accuracy of the standard prognostic model, SII may help discern patients who are likely to benefit from adjuvant intravesical treatments from those who are unlikely to have an oncologic benefit while suffering from unnecessary adverse events. Since BC is an inflammatory disease, SII has tangible potential to improve NMIBC patient care, together with other biomarkers that capture host and tumor factors.

## Supplementary Information

Below is the link to the electronic supplementary material.

Supplementary file1 (PDF 770 KB)

Supplementary file2 (DOCX 21 KB)

## Data Availability

Yes.
