# Supplementary material for: Prognostic value of the systemic immune-inflammation index in non-muscle invasive bladder cancer
Source: World J Urol. 2021 Jun 18;39(12):4355–61. doi: 10.1007/s00345-021-03740-3 (PMC8602174; doi:10.1007/s00345-021-03740-3)
Supplement: Supplementary file 1 — Supplementary file1 (PDF 770 KB) [file 345_2021_3740_MOESM1_ESM.pdf]

Supplementary figure 1.

Kaplan-Meier analysis for recurrence-free survival (RFS) (A), progression-free survival (PFS) (B), overall survival (OS) (C), and cancer-specific survival (CSS) (D) in patients underwent transurethral resection of the bladder (TURB) for non-muscle invasive bladder cancer according to the preoperative systemic immune-inflammation index (SII) level.

Supplementary figure 1A

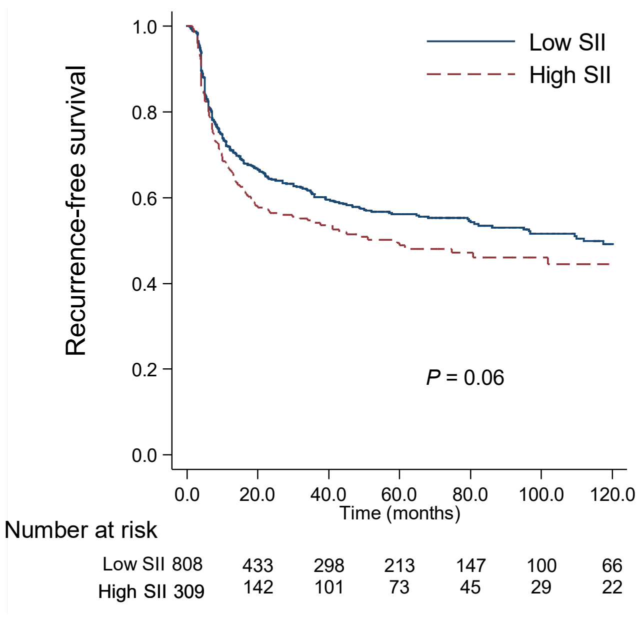

Supplementary figure 1B

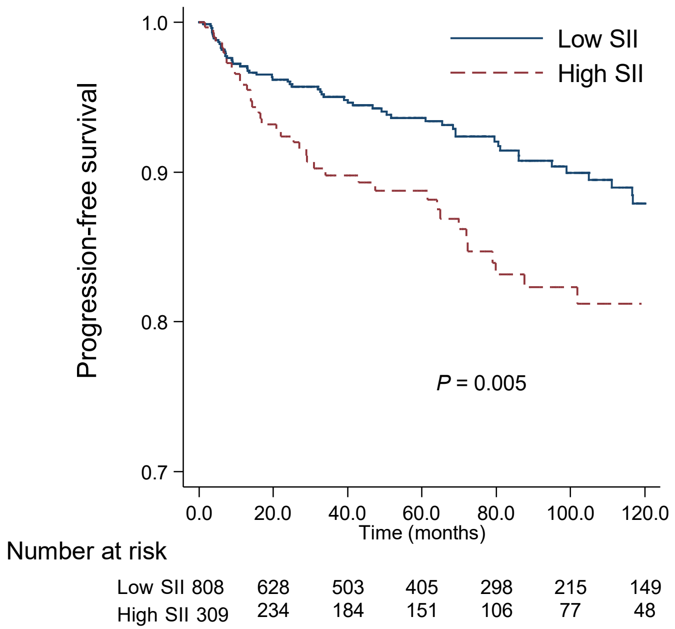

Supplementary figure 1C

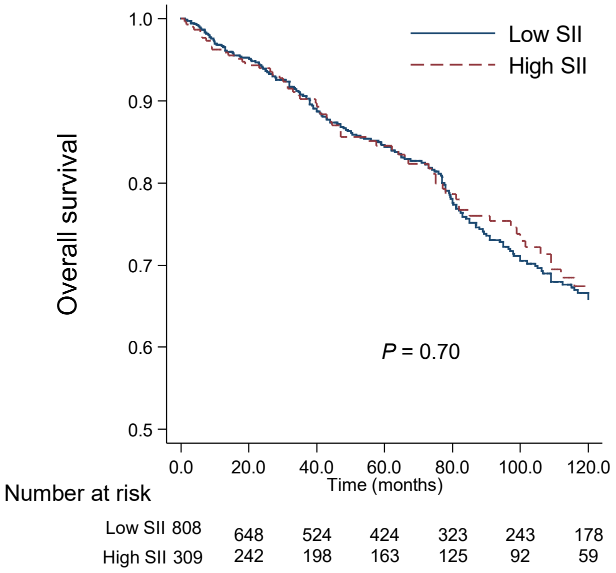

Supplementary figure 1D

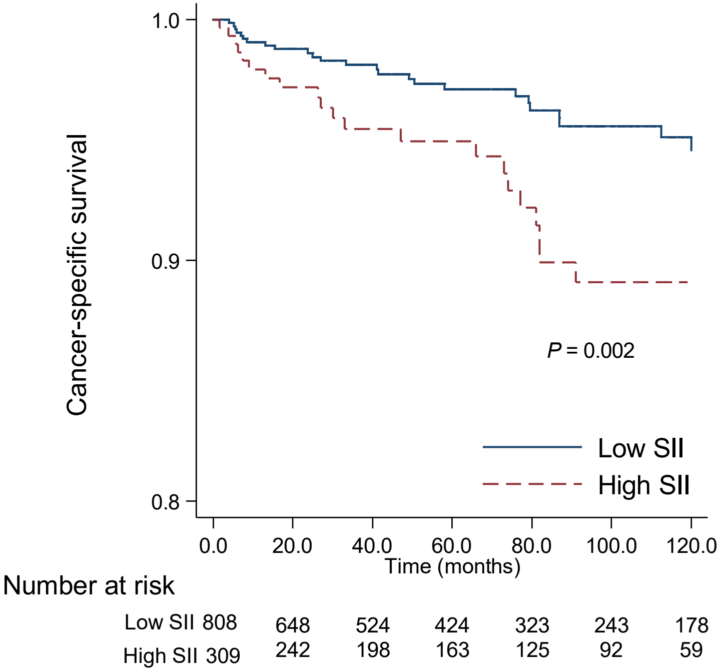

Supplementary figure 2

Decision curve analysis (DCA) for the net-benefit of systemic immune-inflammation index (SII) in the predictive model based on age, sex, tumor size, multifocality, tumor grade, and tumor stage, for recurrence-free survival (RFS) (A), progression-free survival (PFS) (B), overall survival (OS) (C), and cancer-specific survival (CSS) (D) for patients with intermediate-risk.

Supplementary figure 2A

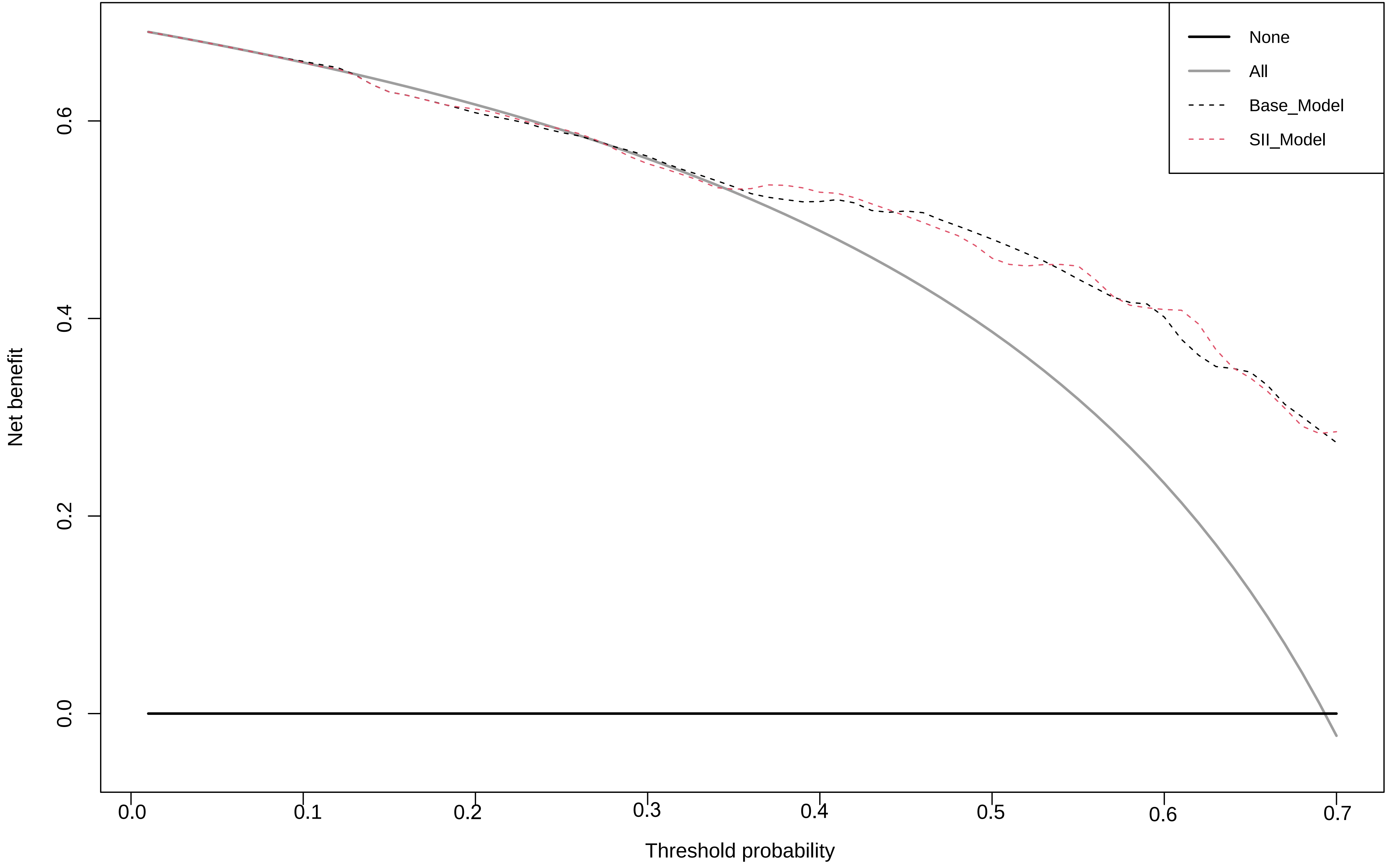

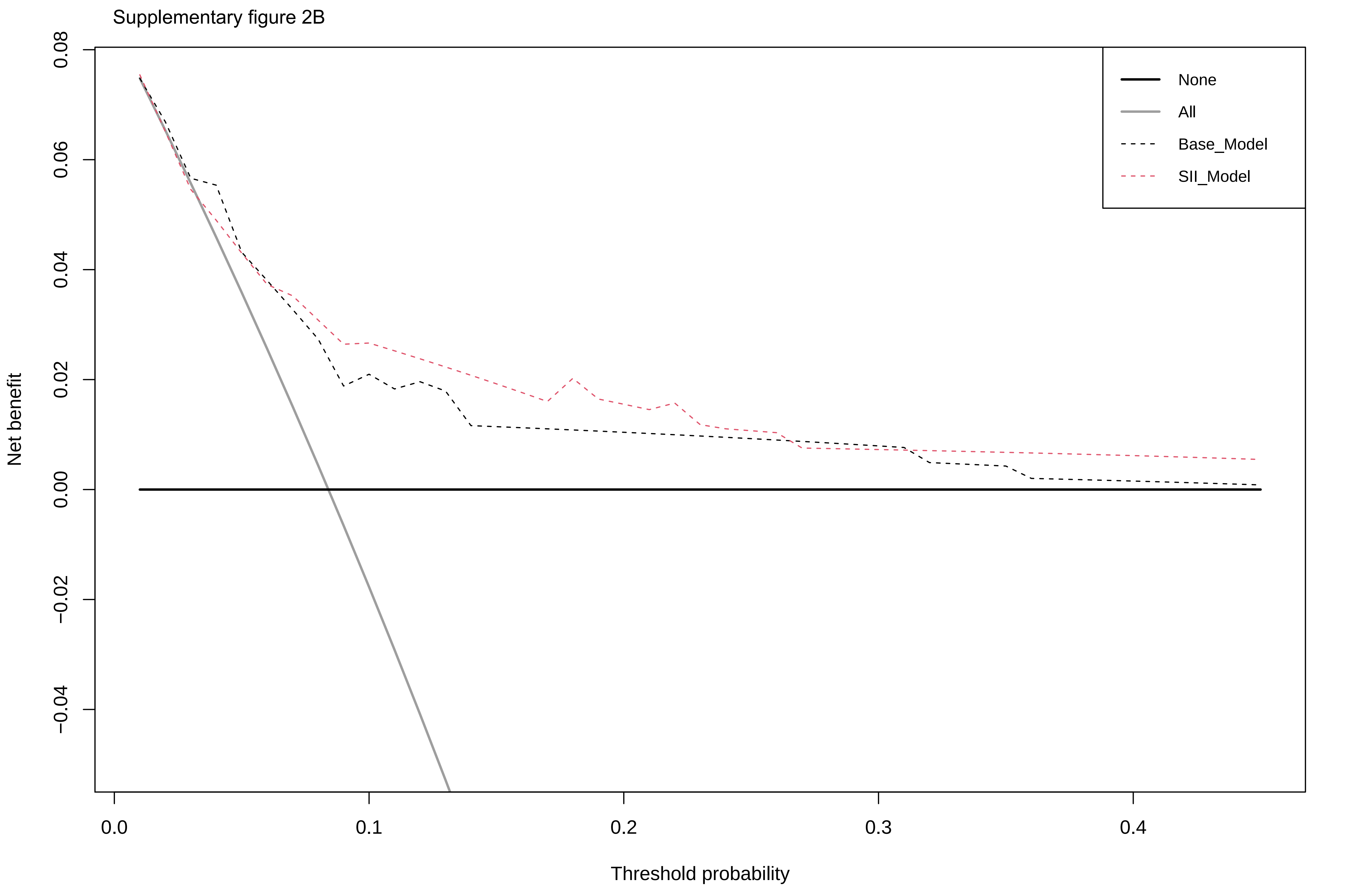

Supplementary figure 2B
Net benefit
Threshold probability
0.08
0.06
0.04
0.02
0.00
−0.02
−0.04
0.0
0.1
0.2
0.3
0.4
None
All
Base_Model
SII_Model

Supplementary figure 2C

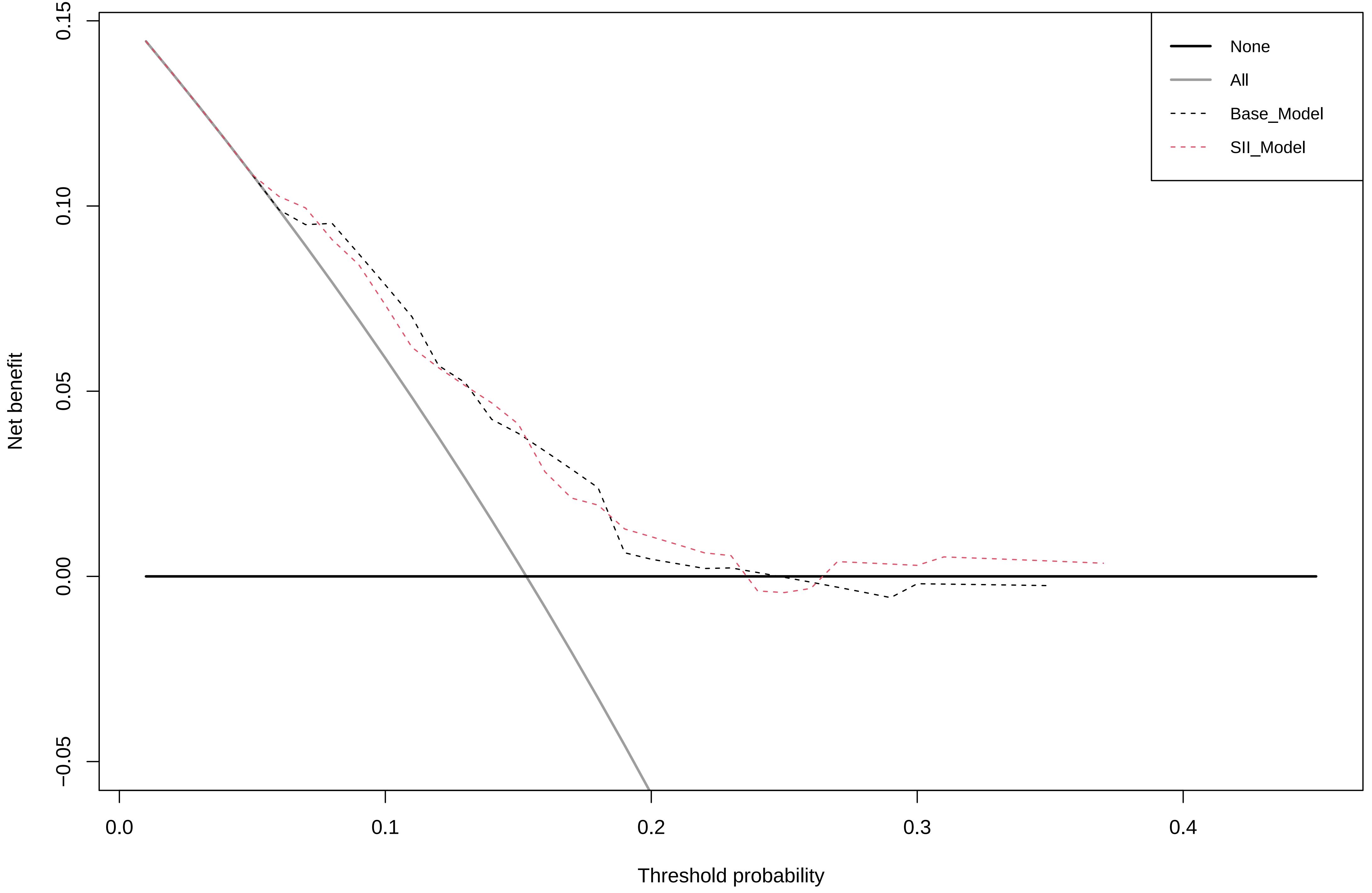

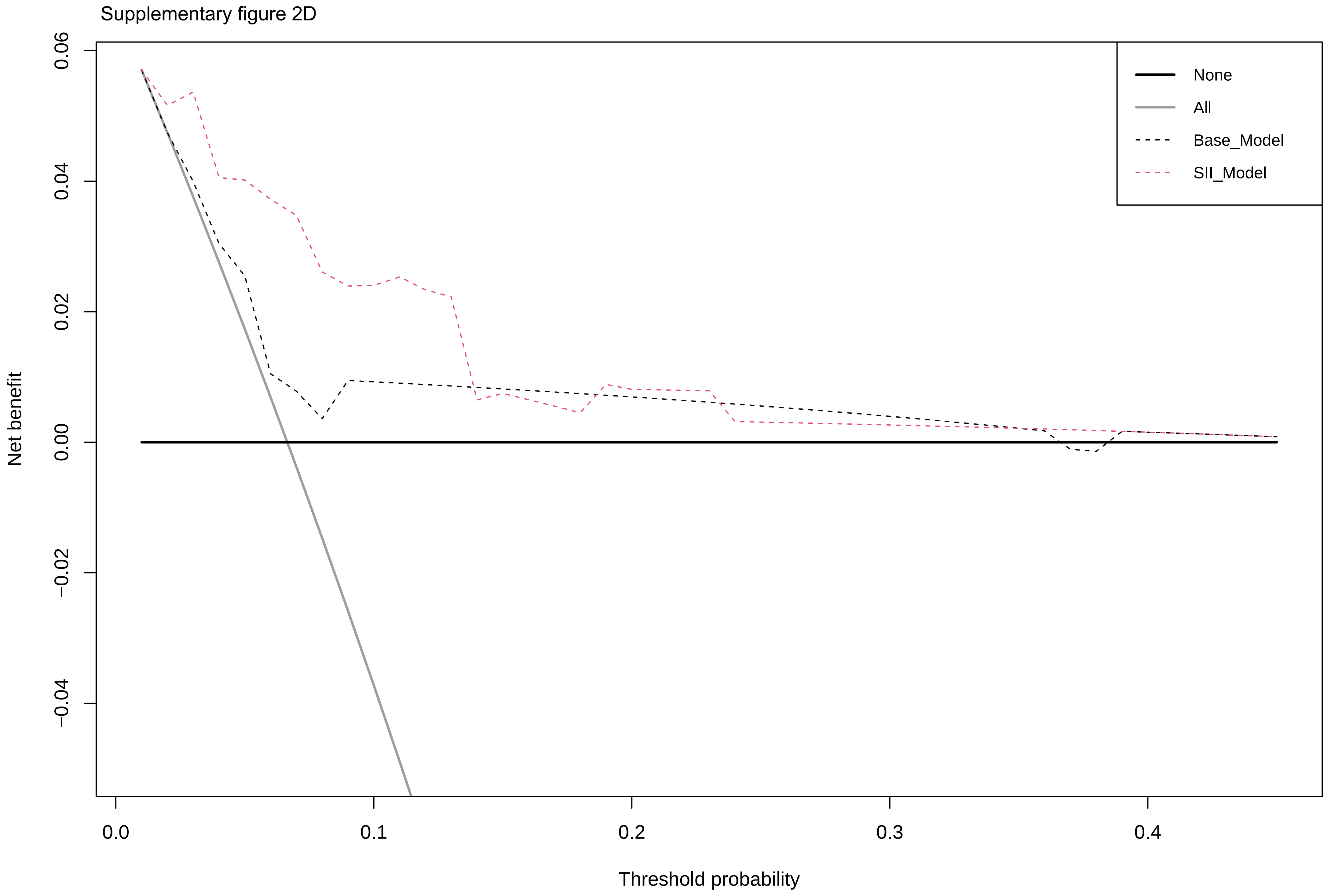
Supplementary figure 2D
Net benefit
0.06
0.04
0.02
0.00
−0.02
−0.04
0.0
0.1
0.2
0.3
0.4
Threshold probability
None
All
Base_Model
SII_Model
