# Supplementary material for: Prognostic value of the systemic immune-inflammation index in non-muscle invasive bladder cancer
Source: World J Urol. 2021 Jun 18;39(12):4355–61. doi: 10.1007/s00345-021-03740-3 (PMC8602174; doi:10.1007/s00345-021-03740-3)
Supplement: Supplementary file 2 — Supplementary file2 (DOCX 21 KB) [file 345_2021_3740_MOESM2_ESM.docx]

| **Supplementary table 1.** Multivariable cox regression analyses in 217 patients with intermediate-risk group substratified by the International Bladder Consultation Group (1-2 factors). | | | | | | | | | | | | | | | |
| --- | --- | --- | --- | --- | --- | --- | --- | --- | --- | --- | --- | --- | --- | --- | --- |
|  | **OS** | | |  | **CSS** | | |  | **PFS** | | |  | **RFS** | | |
|  | Multivariable | | |  | Multivariable | | |  | Multivariable | | |  | Multivariable | | |
|  | HR | (95% CI) | *P* value |  | HR | (95% CI) | *P* value |  | HR | (95% CI) | *P* value |  | HR | (95% CI) | *P* value |
| **Age** (ref. ≤70) |  |  |  |  |  |  |  |  |  |  |  |  |  |  |  |
| >70 | 1.89 | 1.22-2.94 | **0.012** |  | 1.05 | 0.31-3.53 | 0.94 |  | 1.28 | 0.53-3.07 | 0.56 |  | 1.40 | 0.98-2.00 | 0.06 |
| **Sex** | 0.50 | 0.27-0.93 | **0.037** |  | 0.49 | 0.11-2.22 | 0.36 |  | 0.34 | 1.00-1.16 | 0.08 |  | 0.61 | 0.39-0.97 | **0.03** |
| **Grade** (ref. Grade1,2) |  |  |  |  |  |  |  |  |  |  |  |  |  |  |  |
| Grade3 | 1.53 | 0.37-6.34 | 0.74 |  | 8.19 | 1.63-41.2 | **0.01** |  | 11.8 | 3.81-36.5 | **<0.001** |  | 1.98 | 0.78-5.055 | 0.15 |
| **Prior recurrence rate**  (ref. Primary, ≤1 recurrence/yr) | |  |  |  |  |  |  |  |  |  |  |  |  |  |  |
| >1 recurrence/yr |  |  |  |  |  |  |  |  |  |  |  |  | 0.25 | 0.07-0.82 | **0.02** |
| **SII** | 1.56 | 0.97-2.51 | 0.09 |  | 5.15 | 1.72-16.9 | **0.005** |  | 3.71 | 1.62-8.51 | **0.002** |  | 1.36 | 0.92-2.01 | 0.12 |
| C-index without SII | 0.61 | | |  | 0.68 | | |  | 0.62 | | |  | 0.58 | | |
| C-index with SII | 0.63 | | |  | 0.80 | | |  | 0.69 | | |  | 0.59 | | |

*SII* systemic inflammatory index, *OS* overall survival, *CSS* cancer-specific survival, *PFS* progression-free survival, *RFS* recurrence-free survival
